# 

**Published:** 1891-02

**Authors:** 


					﻿Vol. XI.
FEBRUARY, 1891.
No. 2.
’THE
pOMEEOPATHlC
A Monthly Journal of Homoeopathic Materia Medica
and Clinical Medicine.
“ He is a freeman whom truth makes free, and all are slaves beside."
*1 Seek the Truth: come whence it may, cost what it will.”
EDITED AND PUBLISHED BY
WALTER M. JAMES, M. D.,
AND
George H. Clark, m. d.
PHILADELPHIA :
No. 1125 SPRUCE STREET.
iiOifruonsr
ALFRED HEATH <fc CO., Sole Agents for Great Britain,
114 Ebnry Street, Eaton Square.
f$S*Yearly Subscription, $2.50, invariably in advance. Single numbers, 25 cts.
Entered at the Philadelphia Post-Office as Second-Class Hail Matter.
				

